# Thanks to Reviewers 2015

**DOI:** 10.5334/pb.bo

**Published:** 2015-12-31

**Authors:** Editorial Team

**Affiliations:** 1 Psychologica Belgica, Belgium

## Abstract

All manuscripts published in *Psychologica Belgica* have been assessed conscientiously and unselfishly by expert reviewers. The quality of our journal totally depends on their valuable and constructive criticisms to the authors. Both the editors and the authors highly appreciate the input and dedication of all our reviewers. Many thanks.

**Nathalie Aelterman**, Ghent University, Belgium

**Aikaterini – Aliki Michou**, Bilkent University, Turkey

**Jessica Alleva**, Maastricht University, Netherlands

**Carryl Baldwin**, George Mason University, USA

**Christine Bastin**, University of Liège, Belgium

**Moti Benita**, Ben Gurion University of the Negev, Israel

**Raul Berrios**, University of Sheffield, UK

**Wim Beyers**, Ghent University, Belgium

**Filip Boen**, Katholieke Universiteit Leuven, Belgium

**Piet Bracke**, Ghent University, Belgium

**Lieven Brebels**, Katholieke Universiteit Leuven, Belgium

**Bernd Carette**, Ghent University, Belgium

**Fabienne Chetail**, Université Libre de Bruxelles, Belgium

**Elien De Caluwé**, Ghent University, Belgium

**Kathleen De Cuyper**, Katholieke Universiteit Leuven, Belgium

**Jan De Mol**, Université catholique de Louvain, Belgium

**Gert-Jan De Muynck**, Ghent University, Belgium

**Edina Doci**, Vrije Universiteit Brussel, Belgium

**Gesine Dreisbach**, Universität Regensburg, Germany

**Heiner Drenhaus**, Saarland University, Germany

**Martin Edwards**, The university of Sydney, Australia

**Andrew Elliot**, University of Rochester, USA

**Chiedu Eseadi**, University of Nigeria Nsukka, Nigeria

**Mariana Falconier**, Virginia Tech, USA

**Marjolein Feys**, Ghent University, Belgium

**Charlie Frowd**, University of Winchester, UK

**Nicolas Gillet**, Université Francois-Rabelais de Tours, France

**Luc Goossens**, Katholieke Universiteit Leuven, Belgium

**Sharon Goto**, Pomona College, USA

**Leen Haerens**, Ghent University, Belgium

**Michel Hansenne**, University of Liège, Belgium

**Walter Herbranson**, Whitman College, USA

**Kineta Hung**, Hong Kong Baptist University, Hong Kong

**Peter Iserbyt**, Katholieke Universiteit Leuven, Belgium

**Konrad Jankowski**, University of Warsaw, Poland

**Anne Joosten**, Ghent University, Belgium

**Theo Klimstra**, Tilburg University, Netherlands

**Fleur Kraanen**, University of Amsterdam, Netherlands

**Julie Krans**, Katholieke Universiteit Leuven, Belgium

**Leah Lefebvre**, University of Wyoming, USA

**Craig Leth-Steensen**, Carleton University, Canada

**Michael Lewis**, Cardiff University, UK

**Lisa Linnenbrink-Garcia**, University of Michigan, USA

**Koen Luyckx**, Katholieke Universiteit Leuven, Belgium

**Geneviève Mageau**, University of Montreal, Canada

**Lars-Erik Malmberg**, University of Oxford, UK

**Lennia Matos Fernandez**, Pontifical Catholic University of Peru, Peru

**Dora Matzke**, University of Amsterdam, Netherlands

**Anneleen Mortier**, Ghent University, Belgium

**Greta Noordenbos**, Leiden University, Netherlands

**David O’Brien**, Baruch College, USA

**Lauri Oksama**, National Defence University, Finland

**Leen Oris**, Katholieke Universiteit Leuven, Belgium

**Soren Ostergaard**, Aarhus University, Denmark

**Daniele Panizza**, University of Göttingen, Germany

**Thea Peetsma**, University of Amsterdam, Netherlands

**Maria Ranzini**, Université libre de Bruxelles, Belgium

**Catherine Ratelle**, Université Laval, Canada

**Guy Roth**, Ben-Gurion University of the Negev, Israel

**Walter Schaeken**, Katholieke Universiteit Leuven, Belgium

**Rachel Seginer**, University of Haifa, Israel

**Corwin Senko**, State University of New York, USA

**Dirk Smits**, Katholieke Universiteit Leuven, Belgium

**Bart Soenens**, Ghent University, Belgium

**Arnaud Szmalec**, University College London, UK

**Peter Theuns**, Vrije Universiteit Brussel, Belgium

**Elisabet Tubau**, Universitat de Barcelona, Spain

**Jasper Van Assche**, Ghent University, Belgium

**Sara Van Autreve**, Ghent University, Belgium

**Bram Van den Bergh**, Erasmus University, Netherlands

**Hans van der Baan**, University of Amsterdam, Netherlands

**Pieter Van Dessel**, Ghent University, Belgium

**Julie Vandewalle**, Ghent University, Belgium

**Kim Van Durme**, Ghent University, Belgium

**Janne Vanhalst**, Katholieke Universiteit Leuven, Belgium

**Karla Van Leeuwen**, Katholieke Universiteit Leuven, Belgium

**Hubert Van Puyenbroeck**, Vrije Universiteit Brussel, Belgium

**Maarten Vansteenkiste**, Ghent University, Belgium

**Tim Vantilborgh**, Vrije Universiteit Brussel, Belgium

**Julie Verstraeten**, Ghent University, Belgium

**Bart Wille**, Ghent University, Belgium

**Kim Willems**, Vrije Universiteit Brussel, Belgium

**Alex Wood**, University of Stirling, UK

**Ralf Wolfer**, University of Oxford, UK

**Mingming Zhou**, University of Macau, China

